# Lipid Nanoparticle Delivery System for mRNA Encoding B7H3‐redirected Bispecific Antibody Displays Potent Antitumor Effects on Malignant Tumors

**DOI:** 10.1002/advs.202205532

**Published:** 2022-11-20

**Authors:** Cheng Huang, Xing Duan, Jichao Wang, Qingqing Tian, Yangmei Ren, Kepan Chen, Zongliang Zhang, Yuanyou Li, Yunyu Feng, Kunhong Zhong, Yuelong Wang, Liangxue Zhou, Gang Guo, Xiangrong Song, Aiping Tong

**Affiliations:** ^1^ State Key Laboratory of Biotherapy and Cancer Center Research Unit of Gene and Immunotherapy Chinese Academy of Medical Sciences Collaborative Innovation Center of Biotherapy West China Hospital Sichuan University Chengdu Sichuan Province 610041 China; ^2^ Department of Neurosurgery West China Hospital West China Medical School Sichuan University Chengdu Sichuan Province 610041 China; ^3^ Department of Critical Care Medicine and Department of Pancreatic Surgery Frontiers Science Center for Disease‐related Molecular Network State Key Laboratory of Biotherapy and Cancer Center West China Hospital Sichuan University Chengdu Sichuan Province 610213 China

**Keywords:** B7H3, bispecific antibody, cancer, immunotherapy, ionizable lipid nanoparticles, mRNA therapeutic

## Abstract

The therapeutic use of bispecific T‐cell engaging (BiTE) antibodies has shown great potential for treating malignancies. BiTE can simultaneously engage CD3ε on T cells and tumor antigen on cancer cells, thus exerting an effective antitumor effect. Nevertheless, challenges in production, manufacturing, and short serum half‐life of BiTE have dampened some of the promise and impeded the pace of BiTE‐based therapeutics to combat diseases. Nowadays, in vitro‐transcribed mRNA has achieved programmed production, which is more flexible and cost‐effective than the traditional method of producing recombinant antibody. Here, the authors have developed a BiTE‐based mRNA treatment by encapsulating mRNA encoding B7H3×CD3 BiTE into a novel ionizable lipid nanoparticles (LNPs). The authors have found that LNPs have high transfection efficiency, and the hepatosplenic targeting capability of produce high concentrations of BiTE. Above all, a single intravenous injection of BiTE mRNA‐LNPs could achieve high levels of protein expression in vivo and significantly prolonged the half‐life of the BiTE, which can elicit robust and durable antitumor efficacy against hematologic malignancies and melanoma. Therefore, their results suggested that the therapeutic strategy based on mRNA expression of B7H3×CD3 BiTE is of potential research value and has promising clinical application prospects.

## Graphical abstract

In vitro‐transcribed mRNA encoding B7H3×CD3 BiTE is encapsulated in anovel ionizable lipid (IC8) nanoparticles targeting the liver. Compared with recombinant antibody, BiTE mRNA‐loaded LNPs could achieve high levels of protein expression in vivo and significantly prolonged the half‐life of the BiTE, which can elicit robust and durable antitumor efficacy against hematologic malignancies and melanoma.

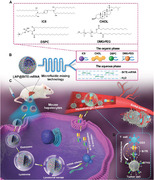

## 1 Introduction

In recent years, BiTE‐based therapy has shown an impressive impact on treating malignancies and other treating cancer. Bispecific antibodies have two antigen‐binding arms, one of which binds to the target antigen and the other to a marker antigen on the effector cell, which activate the effector cell and recruit T cells to form cytolytic synapses with tumor cells.^[^ ^1^, ^2^, ^3^ ^]^ However, a drawback of these immunotherapy approaches against cancer is the need for large amounts of purified BiTE to exert antitumor effects.^[^ ^4^, ^5^, ^6^ ^]^ In addition, several problems are associated with antibody treatment, including high cost, rapid clearance, poor in vivo stability and adverse effects that develop from toxicity.^[^ ^7^, ^8^ ^]^ Nowadays, mRNA has been proven to be a promising method for the continuous expression of antibodies, on account of its simple production process and fast development speed and does not require complex and expensive laboratory infrastructure for recombinant antibody purification, and this significantly affects the amount of BiTE expressed on mRNA, which determines the therapeutic effect of Bi.^[^ ^4^ ^]^

The key to the success of mRNA strategies is to ensure the stabilization of mRNA under physiological conditions and efficient delivery to the target tissue. SciemRNA molecules are easily degraded by Rnase, which is abundant in vitro and in vivo, and the major hurdles in mRNA delivery which is internalization and escape from the endosomes to be localized into the cytosol. As <1/10 000 of delivered mRNA reaches to cytoplasm of recipient cells, rest is degraded or secreted into extracellular vesicles. which is abundant in vitro and in vivo.^[^ ^9^, ^10^ ^]^ Consequently, the mRNA delivery system plays an essential role in stabilizing the mRNA structure, controlling the accessibility to ribosomes and influencing the translational mechanisms. LNPs‐ are the most clinically advanced mRNA delivery system.^[^ ^1^ ^]^ Two LNP‐based SARS‐CoV‐2 mRNA vaccines, BNT162b2 (from Pfizer BioNTech)^[^ ^11^, ^12^ ^]^ and mRNA‐1273 (from Moderna)^[^ ^13^, ^14^ ^]^ have received FDA marketing approval for controlling the SARS‐CoV‐2 pandemic, respectively. which tremendously accelerated the development of mRNA delivery. Recently, mRNA‐1944 (NCT03829384, clinical stage I), which encodes a human IgG antibody connected with Moderna's proprietary LNP technology, was used for antiviral‐miscellaneous vaccines.^[^ ^15^ ^]^ These studies all illustrate the clinical potential of the application of mRNA technologies. Among them, efficient mRNA delivery vectors have become a research hotspot, especially nonviral vectors. C12‐200,^[^ ^16^ ^]^ DLin‐MC3‐DMA,^[^ ^16^ ^]^ SM‐102, ALC‐0315,^[^ ^17^ ^]^ and some other vectors are recognized as the dominant vectors for mRNA delivery in industry. However, all of them have complex synthesis processes, making it difficult to achieve rapid synthesis and quality control. In the field of IVT‐ mRNA as protein replacement therapy,^[^ ^18^, ^19^ ^]^ it is critical to establish an efficient and low toxicity delivery (vector) system that facilitates mRNA to targeted tissues and organs.^[^ ^20^ ^]^ Thus, in this work, we prepared a novel ionizable lipid IC8 through a simple one‐step reaction, and constructed a delivery system for the mRNA encoding the B7H3×CD3 BiTE.

B7 homolog 3 protein (B7H3), also known as CD276, is a type I transmembrane protein that belongs to the B7 family of immune checkpoint proteins.^[^ ^21^ ^]^ Aberrant expression of B7H3 has been found in a variety of cancers, such as melanoma, head and neck cancer, lung adenocarcinoma, craniopharyngioma, neuroblastoma, glioma, ovarian cancer, pancreatic cancer and acute myeloid leukemia (AML).^[^ ^22^, ^23^, ^24^ ^]^ However, its expression is absent or low in normal tissues.^[^ ^25^, ^26^, ^27^ ^]^ The expression of B7H3 on the surface of tumor cells has become a driver for tumor growth caused by tumor escape from immune cell pursuit.^[^ ^28^ ^]^ Blockade of B7H3 by chimeric antigen receptor T cells.^[^ ^23^ ^]^ or monoclonal as well as (BsAbs) has shown ideal outcomes in hematologic and solid tumors. In preclinical studies. ATG027, a B7H3/PD‐L1 bispecific antibody designed by Antengene, is currently being used in preclinical studies for the treatment of hematologic malignancies and solid tumors. ATG027 is also planned to submit IND/CTA applications in 2022.^[^ ^23^ ^]^ Therefore, overexpression of B7H3 is considered an attractive biomarker and target for multiple cancer immunotherapy approaches.^[^ ^29^ ^]^

Here, we show that a novel and liver‐targeted ionizable lipid nanoparticle delivery system for mRNA encoding B7H3×CD3 BiTE exerts potent antitumor activity against hematologic and solid tumors compared with purified recombinant BiTE. Our results demonstrated that the nucleoside‐modified mRNA‐LNPs platform is a safe, simple, and efficient alternative to therapeutic protein delivery with potential clinical application to any monoclonal or bispecific antibody with extension to any therapeutic protein.

## 2 Results

### 2.1 Analysis of B7H3 Expression Based on Online Database and Clinical Samples of AML and Melanoma

**Figure 1:**
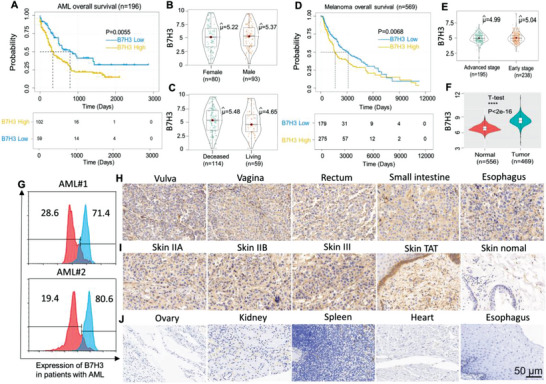
Expression of the costimulatory molecule B7H3 in‐AML and human melanoma samples. A) Relationship between B7H3 expression and the overall survival of patients with AML (log‐rank test, *p*  =  0.0055). B) The mRNA expression of B7H3 in AML male and female samples analyzed with the TCGA database. (Female vs Male, *p* > 0.05). C) The mRNA expression of B7H3 in deceased and living AML patients analyzed with the TCGA database. (*p* > 0.05). D) The relationship between B7H3 expression and the overall survival of patients with melanoma (log‐rank test, *p* = 0.0068). E) The mRNA expression of B7H3 in advanced stage and early‐stage tumor tissue samples analyzed with the TCGA database. (*p* > 0.05). F) The mRNA expression of B7H3 in normal and tumor melanoma tissue samples analyzed with the GTEX and TCGA databases respectively (p**** =1.8e‐16< ). G) the expression of B7H3 in AML patient samples was evaluated by FACS. H) TMAs of human melanoma organs/anatomic site were stained for IHC to detect the expression of B7H3. I) TMAs of human melanoma skin tissue stage (IIA–III), human ovarian tumor‐adjacent (TAA) and normal skin tissues were stained for IHC to detect the expression of B7H3. J) TMAs of human normal tissues were stained for IHC to detect the expression of B7H3. Scale bars, 50 µm.

First, we set out to perform bioinformatic analyses on the AML and melanoma patient databases. We analyzed the association between B7H3 expression and AML patient overall survival using the KM‐plotter database (http://kmplot.com) (**Figure** **1**A). Survival analysis showed that the low‐risk group had a better overall survival than the high‐risk group. However, no significant differences were observed among the four groups (femal vs male, deceased patients vs living patients) (Figure 1B,C). In addition, three studies showed the differential expression of B7H3 in AML samples compared with normal samples. Darker red indicates higher B7H3 expression. (Figure S1A, Supporting Information). For the human melanoma patients, similar trends from Figure 1A were observed for overall survival (Figure 1D). Then, the mRNA expression level of B7H3 was tested at different clinical stages (stages 0–iv). However, the B7H3 mRNA expression level was not significantly different among different clinical stages (Figure S1B, Supporting Information). Likewise, no obvious difference could be identified between the tumor advanced stage and early stage tissue samples (Figure 1E). Moreover, the data showed that B7H3 mRNA expression in melanoma cancer tissues was significantly higher than that in matched normal skin tissues (Figure 1F). Finally, five studies showed the differential expression of B7H3 in melanoma tissues compared with normal tissues. Darker red indicates higher B7H3 expression. (Figure S1C, Supporting Information). As a result, we considered B7H3 as a biosafe clinical target.

### 2.2 Frequent Expression of B7H3 in AML and Melanoma Clinical Samples and Various Human Tumor Cell Lines

To further assess the expression of B7H3, expression of B7H3 was examined in the AML clinical samples were examined by flow cytometry (FACS) using anti‐B7H3 antibody as the primary antibody. As shown in Figure 1G, the B7H3 expression levels of two patients with monocytic/myelomonocytic AML, determined by FACS as positive proportion values, were 71.4% and 80.6%, respectively. Furthermore, immunohistochemistry (IHC) was used to analyze B7H3 expression on 80 paraffin‐embedded malignant melanomas with skin tissue and paired paracarcinoma tissues on tissue microarrays (TMAs) (Figure 1H,I). Malignant melanoma with normal skin tissue microarray, containing 40 cases of skin malignant melanoma, 20 adjacent normal skin tissue and 20 normal organs and skin tissue. The expression of B7H3 protein was markedly higher in malignant melanoma with skin tissue than in tumor‐adjacent normal tissues and normal tissues. Compared with normal tissues (Figure 1J), a stronger increase in B7H3 protein expression was observed in tumor‐adjacent normal tissues. Next, we also determined the B7H3 protein expression level in various cancer cell lines by using FACS and immunofluorescence (Figure S2A–F, Supporting Information). These data showed that the majority of tumor cells had a high level of B7H3 expression, with very few (hematologic tumor cell lines) exceptions. Together, these results indicate that B7H3 may serve as a promising clinical target for solid hematologic and tumor treatment.

### 2.3 Characterization and the In Vitro Antitumor Effects of B7H3×CD3 BiTE

**Figure 2:**
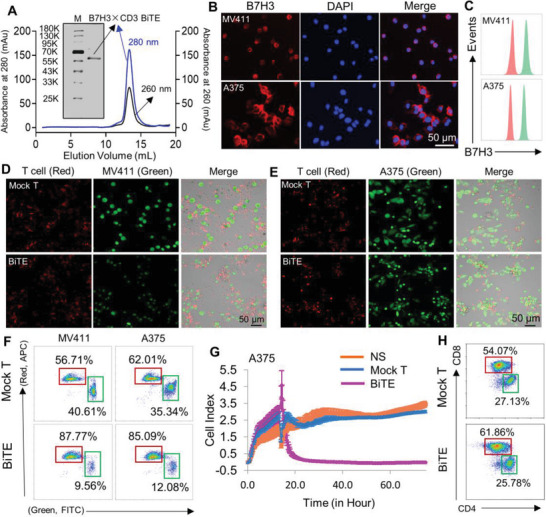
Construction and cytotoxicity of anti‐B7H3×CD3 BiTE in vitro. A) SDS PAGE and gel filtration chromatograph of anti‐B7H3×CD3 BiTE. M: marker. B,C) Immunofluorescence staining and FACS analysis of the expression of B7H3 in MV411 and A375 cells. Scale bars, 50 µm. Cells were incubated with human B7H3 antibody (red) or its corresponding isotype control (green). D–F) The morphology and result of tumor cell lysis were analyzed using confocal microscopy and FACS respectively. T cells with the BiTE (BiTE) and cells without BiTE (Mock T) are experimental and control groups, respectively. Scale bars, 50 µm. G) Target cell (A375) survival curves were recorded by the xCELLigence real‐time cell analyzer. H) Dot plot diagram of FACS showing the CD4^+^ and CD8^+^ percentages of human T cells after 5 µg mL^−1^ B7H3×CD3 BiTE treatment for 24 h.

The B7H3‐specific single‐chain antibody fragment (scFv) and CD3‐specific scFv were linked by a 5‐amino‐acid (G4S) linker to form a recombinant single‐chain BiTE. For recombinant BiTE expression in mammalian cell lines, cDNA encoding the CD3‐specific scFv and B7H3‐specific scFv (J42‐scFv) were subcloned into a eukaryotic expression vector with a His tag at the C‐terminal to facilitate protein purification. **Figure** **2**A shows the SDS‐PAGE analysis of purified B7H3×CD3 BiTE by gel filtration chromatography on a Superdex 200 Increase 10/300GL column. The data showed that both cell lines had a high level of B7H3 expression in the MV411 and A375 cancer cell lines (Figure 2B,C). To directly observe and measure the tumor killing effects of BiTE on tumor cells, we performed an in vitro tumor killing assay in cocultured BiTE with MV411, THP‐1, A375, and SKOV3 tumor cells. For all target cells, more T‐cell clusters and cancer cell lysis were observed in the BiTE treatment group (Figure 2D,E and Figure S3A,B, Supporting Information). When BiTE‐mediated lysis of tumor cells, the representative FACS plots are shown in Figure 2F. Both demonstrated the in vitro antitumor effects of BiTE. To further examine whether BiTE could engage T cells to kill A375 tumor cells, a real‐time in vitro tumor‐killing assay was performed. The most significant lytic effect was observed in the BiTE treatment group. However, no obvious cell‐killing effect was observed in growth and proliferation between mock T cells and the control group (Figure 2G). BiTE‐T cells caused 95% cell death in target cells (tumor cells),as shown in Figure S4A, Supporting Information. Increased secretion of interferon *γ* (IFN‐*γ*), interleukin (IL)‐2, and tumor necrosis factor *α* (TNF‐*α*) was measured in B7H3‐redirected BiTE‐T cells cocultured with B7H3‐positive tumor cells (A375, MV411, SKOV3, and THP‐1) (Figure S4B–D, Supporting Information). Before the in vitro and in vivo antitumor assays, the ratio of CD4^+^/CD8^+^ human T cells stimulated by BiTE (5 µg mL^−1^) was analyzed by FACS. After stimulation for 24 h, there was no evident changes in the CD4^+^/CD8^+^ ratio between the BiTE and control group (Figure 2H).

### 2.4 Characterization of Composite Ionizable Lipid Nanoparticles

**Figure 3:**
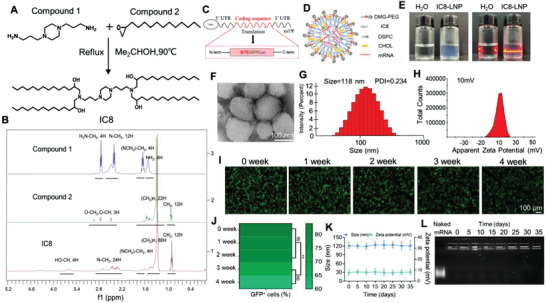
Characterization and physicochemical properties of the designed LNP@GFP‐mRNA. A) Schematic illustration of IC8 synthesis. B) The structure of IC8 was characterized by using the nuclear magnetic technique. C) In vitro mRNA was prepared by an in vitro transcription system. D) Schematic diagram showing the components and 2D structure of LNP@GFP‐mRNA. E) The Tyndall effect of LNP@GFP‐mRNA. F) Representative TEM images of LNP@GFP‐mRNA. Scale bars 100 nm. Weight ratios of 1:15 (mRNA to IC8) were used in LNP synthesis. G) Size distribution of the LNP@GFP‐mRNA determined by the dynamic laser scanning method. H) Apparent zeta potential of LNP@GFP‐mRNA. I) Transfection assay to investigate the mRNA loading stability of LNP from week zero to week fourth stored at 4 °C. Scale bars 100 µm. J) GFP quantitative analysis of LNP@GFP‐mRNA in 293T cells at the indicated times. (mean ± SD, *n* = 3). **p < 0.01 K) The variation in particle size and zeta potential of LNP@GFP‐mRNA after 35 days of storage at 4 °C. L) mRNA loading stability by LNPs was evaluated by a gel retardation assay.

The mRNA delivery lipid IC8 was synthesized based on the standard synthetic procedures described in Section 5 (**Figure** **3**A). 1H NMR (400 MHz, CDCL3) of IC8 is shown in Figure 3B, and its NMR data are as follows: 1H NMR (400 MHz, CDCl3) *δ* (ppm) = 4.61–3.77 (m, 4H), 3.73–3.49 (m, 4H), 2.91–2.14 (m, 24H), 1.68–1.53 (m, 4H), 1.50–1.38 (m, 8H), 1.34–1.23 (s, 88H), 0.88 (t, *J* = 6.8, 12H). The NMR data of compound 1 are as follows: 1H NMR (400 MHz, CDCl3) *δ* = 2.75 (t, *J* = 6.7, 1H), 2.42 (m, *J* = 23.8, 16.2, 3H), 1.75–1.58 (m, 1H), 1.51 (s, 1H). The NMR data of compound 2 are as follows: 1H NMR (400 MHz, CDCl3) *δ* = 2.99–2.88 (m, 1H), 2.82–2.69 (m, 1H), 2.48 (dd, *J* = 4.7, 2.1, 1H), 1.66–1.20 (m, 22H), 0.90 (t, *J* = 6.2, 3H). Figure 3C shows some structural elements of IVT‐mRNA, including the 5′ cap1, 3′ poly(A) tail, protein‐coding sequence (BiTE/GFP/Luciferase), N1‐methyl‐pseudouridine (m1Ψ) modification and 5′ and 3′ untranslated regions (UTRs). The expression plasmid map of B7H3×CD3 BiTE, GFP and luciferase (Luc) (Figure S5, Supporting Information). Next, Figure 3D clearly represents the constituent and the internal structure of the LNP@GFP‐mRNA in more detail. mRNA can be encapsulated into LNPs via electrostatic attraction with the cationic head groups of IC8. An obvious tyndall effect was observed in the LNP solutions after exposure to UV light (right), as shown in Figure 3E. From the obtained TEM photograph of LNPs, we could observe not only a spherical shape but also the curved thread‐like structures of the LNPs and visualize multiple overlapped thread regions (Figure 3F). The LNPs were characterized regarding the mean particle size, polydispersity index (PDI), and zeta potential using a Zetasizer. The three parameters were 118 nm ± 3.95 nm, 0.234 ± 0.12 and 10 ± 1.14 mV respectively (Figure 3G,H). In addition, the transfection ability of LNPs was observed by analyzing the transfection images of LNP@GFP‐mRNA from the zeroth week to the fourth week at room temperature. As shown, we found that GFP mRNA‐LNPs maintained some degree of transfection ability with the extension of LNP@GFP‐mRNA storage time (Figure 3I,J). Finally, after 35 days of storage at 4 °C, the mean particle diameter and zeta potential of the mRNA‐LNPs did not change significantly (Figure 3L). The results above suggested the stability of the structure of the mRNA‐LNPs to some extent.

### 2.5 Endosomal/Lysosomal Escape and Transfection Assay of LNP@mRNA

**Figure 4:**
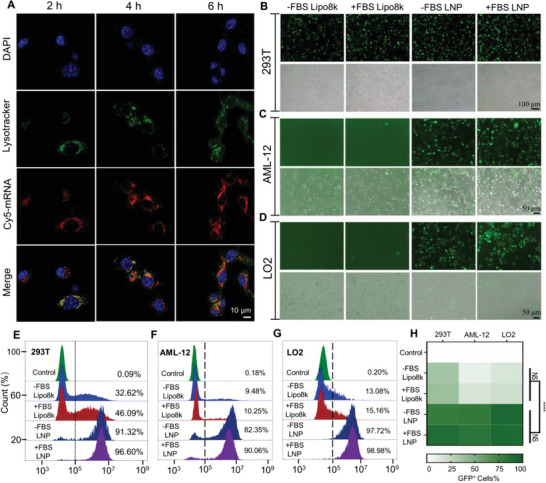
Subcellular colocalization and transfection efficiency. A) CLSM images show the lysosome escape of LNP@ CY5‐mRNA in AML12 cells after 2, 4, and 6 h of incubation. The bar represents 10 µm. B–D) Graphs illustrating B) GFP expression in 293T cell lines, C) mouse normal hepatic cells (AML‐12), and D) human normal liver cells (LO2) under the indicated conditions. Scale bars, 100 and 50 µm, respectively. E–H) FACS and quantitative analysis of GFP expression in the three types of cells mentioned above. NS: no significance, ****p < 0.0001

The internalized LNP@CY5‐mRNA complex must evade endosome or lysosome to function. Therefore, the lysosome escape ability of LNP@CY5‐mRNA was investigated by the confocal laser scanning microscopy (CLSM). the CLSM analysis (**Figure** **4**A) showed that red spots were observed within the AML‐12 cells and that LNP@CY5‐mRNA mainly colocalized with the LysoTracker green stained organelles after 1–2 h of incubation. Subsequently, the separation of the green and red fluorescence spots was more significant for 4–6 h, suggesting that LNP@CY5‐mRNA could efficiently escape from the endosomes or early lysosomes to the cytoplasm. Subsequently, the transfection efficiency of the liposomal formulation was compared to that of the commercially available cationic transfection reagent Lipofectamine 8000. Based on fluorescence microscopy, we unambiguously observed a strong green fluorescence enrichment of these three cell lines (293T AML‐12, and LO2) in the LNP GFP‐mRNA treatment group (Figure 4B–D). Meanwhile, the GFP green fluorescence intensity was analyzed by FACS after the same treatment in the three cell lines. Figure 4E–G shows a relatively good transfection efficiency compared to Lipofectamine 8000. Similarly, the LNP@GFP‐mRNA groups had a significant increase in the relative fluorescence intensity of GFP compared to the positive control. Finally, we found that media containing 10% serum or 0% serum did not affect the transfection efficiency (Figure 4H).

### 2.6 Biodistribution of LNP@Luc‐mRNA and Kinetics of B7H3×CD3 BiTE Production In Vivo

**Figure 5:**
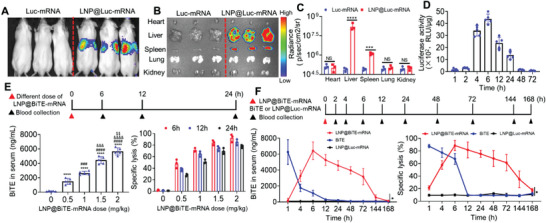
Biodistribution of LNP@Luc‐mRNA and protein expression of B7H3×CD3 BiTE in vivo. A) In vivo transfection of LNP@Luc‐mRNA after i.v. injected LNP@ Luc‐mRNA. B) Ex vivo imaging of organs and C) quantitative analysis of luminescence were recorded 6 h after i.v. injection of LNP@Luc‐mRNA. *n* = 3. D) Quantification of luciferase activity in the liver tissue at different time points after the injection of LNP@Luc‐mRNA (Luc‐mRNA: 1.5 mg kg^−1^, i.v.) measured by a luciferase assay kit. *n* = 5 biologically independent samples. E) Concentration (Cp) (left) and ex vivo cytotoxicity (right) of endogenously translated B7H3×CD3 BiTE in NSG mouse serum after i.v. injection of LNP@BiTE‐mRNA at different times and doses. Mean ± SD. ****p < 0.0001 versus the 0 mg kg^−1^ group. ^###^ *p* < 0.001 and ^####^ *p* < 0.0001 versus the 0.5 mg kg^−1^ group. ^&&&^ *p* < 0.001 and ^&&&&^ *p* < 0.0001 versus the 1 mg kg^−1^ group. ^$$^ *p* < 0.01 versus the 1.5 mg kg^−1^ group. F) Pharmacokinetics (left) and ex vivo cytotoxicity (right) of endogenously translated B7H3×CD3 BiTE in the plasma of NSG mice that were i.v. injected with 30 µg (1.5 mg kg^−1^) of LNP@Luc‐mRNA (negative control), 120 µg (6 mg kg^−1^) B7H3×CD3 BiTE or 30 µg (1.5 mg kg^−1^) of LNP@BiTE‐mRNA. *n* = 3. Data are presented as the means ± s.d. *p < 0.05.

To evaluate the liver‐targeted delivery effect of LNP@Luc‐mRNA in vivo, live fluorescence images were collected at 6 h after intravenous injection of LNP@Luc‐mRNA and showed a strong fluorescent signal in the liver sites (**Figure** **5**A). Meanwhile, in vitro fluorescence images of isolated organs were collected at 6 h postinjection. The transfection efficacy was highest for liver tissue, followed by spleen tissue and lowest in other organs, as demonstrated by Figure 5B. Quantitative analysis of Luc fluorescence intensity confirmed that compared to controls, liver tissues from the Luc‐mRNA LNP treatment group had significant changes. Of interest, Luc fluorescence levels in the spleen were improved to different  degrees after intravenousLY (i.v.) administration of LNP@Luc‐mRNA (Figure 5C). In addition, a time course biodistribution of the luciferase activity was performed after i.v. administration of LNP@Luc‐mRNA. We found peak luciferase activity at 6 h after intravenous injection (Figure 5D). To evaluate whether BiTE mRNA is capable of producing protein in vivo, mice were treated with increasing amounts of 0, 0.5, 1, 1.5, or 2 mg kg^−1^ (based on encapsulated BiTE mRNA amount) LNP (*n* = 5). All doses were well tolerated, and no adverse events were observed. As shown in Figure 5E (left), we found a dose‐dependent increase in BiTE protein levels in mouse serum 24 h after administration. Upon injection with 1.5 and 2 mg kg^−1^ BiTE mRNA, an ≈4 and 6 µg mL^−1^ BiTE serum was produced. The in vitro cytotoxic activity increased with increasing BiTE mRNA dose and reached maximal killing effect at 6 h. Of note, there was strong and sustained in vitro cytotoxic activity at 12 and 24 h after LNP@BiTE‐mRNA injection (Figure 5E, right). T cells and MV411 cells (effector‐to‐target,E:T = 5:1) were cocultured with 10% plasma per 100 µL of total assay volume. Moreover, to evaluate whether B7H3×CD3 BiTE generated in vivo in mice shows therapeutic efficacy equivalent to that of the purified recombinant BiTE counterparts, we measured BiTE levels in mouse serum after i.v. administration is shown in Figure 5F. Plasma levels of B7H3×CD3 BiTE endogenously translated from the administered LNP@BiTE‐mRNA peaked within 6 h and were sustained over several days (Figure 5F, left). For the LNP@BiTE‐mRNA treatment group, the following B7H3×CD3 BiTE parmacokinetic parameters are shown: the maximal BiTE concentration in the plasma (6.455 ± 0.824 µg mL^−1^) was observed 6 h after injection, followed by a decrease yet detectable measurement until day 4. The total BiTE level over time (area under the curve) was ≈146.6 h µg mL^−1^. The half‐life of BiTE expression after administration of LNP@BiTE‐mRNA was ≈73 h. Compared to the recombinant BiTE treatment group, administration of LNP@BiTE‐mRNA significantly prolonged the BiTE half‐life (≈2 h). The detailed results are also provided in Table S1, Supporting Information. Accordingly, the in vitro cytotoxic activity of plasma from the treated mouse exerted a maximum lysis of 90% at 6 h and remained above the half‐maximal level for up to 4 d after injection (Figure 5F, right).

### 2.7 In Vivo Antitumor Effects of LNP@BiTE‐mRNA in Human Hematological Tumor Xenograft Models

**Figure 6:**
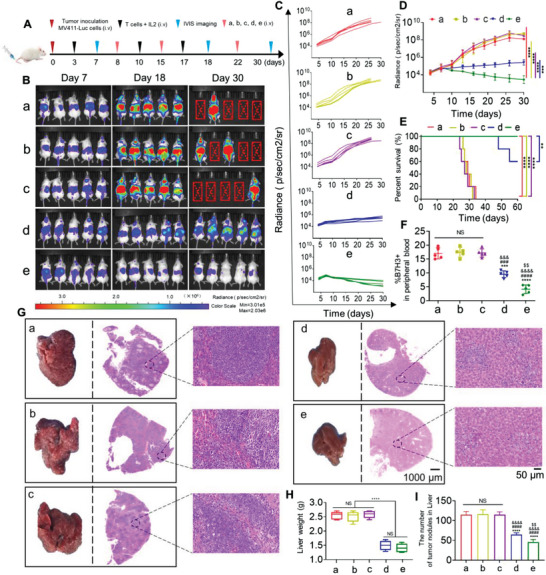
In vivo antitumor effects of LNP@BiTE‐mRNA in human hematological tumor xenograft models. A) Schematic diagram of the immunotherapy regimen. B) Representative tumor growth was shown in vivo by bioluminescence imaging using IVIS 200 at 7, 18, and 30 days after implantation. Laser power density, mean ± SD, 2 W cm^−2^. *n*  = 5. C,D) Individual flux or tumor total data (in p/s) were calculated using living image software. ***p < 0.001 and ****p < 0.0001 compared to (e) at 30 days. E) Survival curves of mice given different treatments. **p < 0.01 and ****p < 0.0001 compared to (e). *n*  = 5. F) B7H3‐positive MV411 cells in peripheral blood were detected by using FACS on day 22 after tumor inoculation. ***p < 0.001 and ****p < 0.0001 versus the group of (a). ^###^ *p* < 0.001 and ^####^ *p* < 0.0001 versus the group of (b). ^&&&^ *p* < 0.001 and ^&&&&^ *p* < 0.0001 versus the group of (c). ^$$^ *p* < 0.01 versus the group of (d). *n*  = 5. G) Liver photographs and H&E staining of MV411 tumor‐bearing mice on day 30 after the indicated treatments. Scare bars, 50 and 1000 µm respectively. H,I). Statistical analysis of the liver weight and number of tumor nodules from the liver was performed quantitatively. ****p < 0.0001. *n*  = 5. ****p < 0.0001 versus the group of (a). ^####^ *p* < 0.0001 versus the group of (b). ^&&&&^ *p* < 0.0001 versus the group of (c). ^$$^ *p* < 0.001 versus the group of (d). (a) Normal saline + T cell, (b) 22 mg kg^−1^ IC8‐LNP + T cell, (c) 1.5 mg kg^−1^ BiTE mRNA + T cell, (d) 6 mg kg^−1^ BiTE + T cell, (e) 1.5 mg/kg LNP@BiTE‐mRNA + T cell.

To assess the antitumor efficacy of LNP@BiTE‐mRNA in vivo, LNP@BiTE‐mRNA was i.v. administered to MV411‐Luc xenograft tumor‐bearing NSG mice in a 60‐day trial. Schematic diagram showing the in vivo treatment program (**Figure** **6**A). We monitored tumor growth by bioluminescence at 7, 18, and 30 days (Figure 6B). mice in the normal saline, IC8‐LNP and BiTE‐mRNA groups showed rapid progression of tumors and most died ≈26 days after tumor inoculation. In contrast, the weakest fluorescence signal of tumors was observed in mice treated with LNP@BiTE‐mRNA, followed by tumors treated with BiTE. Obviously, the tumor individual (Figure 6C) or total bioluminescence (Figure 6D) intensity also showed the same trend compared with the tumor bioluminescence imaging graph. Meanwhile, none of the mice died in the LNP@BiTE‐mRNA group during the trial. This led to a significant survival advantage compared with mice in the other treatment groups (Figure 6E). The numbers of B7H3‐positive MV411 cells in peripheral blood in the LNP@BiTE‐mRNA group were significantly decreased, with a lower index of ≈10.2 ± 2.14% compared with the BiTE group (21.2 ± 2.23%) (Figure 6F). In addition, the liver tissue was collected and photographed (Figure 6G and Figure S6, Supporting Information).We subsequently performed a preliminary evaluation of the in vivo safety of tumor‐bearing mice by H&E staining, we found that the structure of livers in the normal saline. IC8‐LNP and BiTE mRNA treatment groups had significant pathological changes. In the three aforementioned groups, the MV411 tumor cells were highly surface‐enriched for livers and formed many small white dots during the animal trials. In contrast, there was a slight degree of tumor metastasis, normal mouse liver weight and the least amount of tumor nodules in the liver from the BiTE and LNP@BiTE‐mRNA treatment groups (Figure 6G–I). Finally, microscopic observations did not find any lesions in the heart, spleen, kidney or lung in tumor‐bearing mice from the LNP@BiTE‐mRNA treatment groups (Figure S7, Supporting Information).

### 2.8 In Vivo Antitumor Effects of LNP@BiTE‐mRNA in Human Melanoma Subcutaneous Tumor Xenograft Models

**Figure 7:**
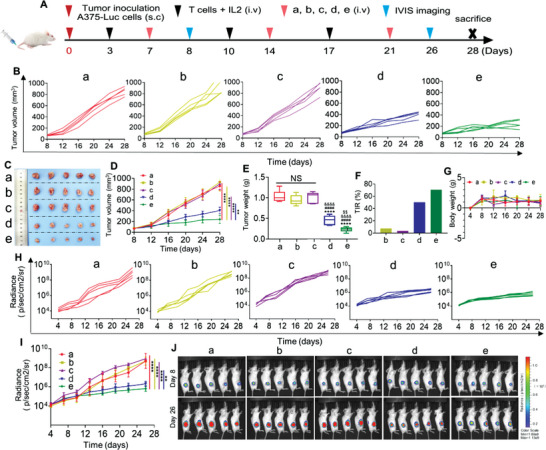
In vivo antitumor effects of BiTE mRNA‐LNPs in human melanoma subcutaneous tumor xenograft models. A) Schematic diagram of the immunotherapy regimen. B) Tumor growth of each mouse in different groups. C) Photographs of A375 tumor‐bearing mouse on day 28 after the indicated treatments. D–G) Growth of D) tumor volume, E) tumor weight, F) the tumor inhibition rate, and G) body weight change in different groups of mouse. *n*  = 5.**p < 0.01 and ****p  < 0.0001 compared to (e) at 28 days. ****p < 0.0001 versus the group of (a). ^####^ *p* < 0.0001 versus the group of (b). ^&&&&^ *p* < 0.0001 versus the group of (c). ^$$^ *p* < 0.001 versus the group of (d). H,I) Tumor individual or total flux data (in p/s) were calculated using living image software. *n*  = 5.**p < 0.01 and ****p < 0.0001 compared to (e) at 26 days. J) The representative tumor growth was shown in vivo by bioluminescence imaging using IVIS 200 at 8 and 26 days after implantation. Laser power density, mean ± SD, 2 W cm^−2^ (*n*  = 5). (a) Normal saline + T cell, (b) 22 mg kg^−1^ IC8‐LNPs + T cell, (c) 1.5 mg kg^−1^ BiTE mRNA + T cell, (d) 6 mg kg^−1^ BiTE + T cell, (e) 1.5 mg kg^−1^ BiTE mRNA‐LNPs + T cell.

To evaluate the response of LNP@BiTE‐mRNA to treatment in solid tumors, a melanoma A375 tumor‐bearing NSG mouse model was established. A schematic diagram of the immunotherapy regimen is shown in **Figure** **7**A. As presented in Figure 7B–D. There were no significant differences in the volume of the tumor among the normal saline, IC8‐LNP and BiTE mRNA treatment groups. The BiTE and LNP@BiTE‐mRNA treatments exerted more obvious inhibitory effects on the tumor volumes than the other treatments. Similar conclusions were reached after assessing tumor weight (Figure 7E). Likewise, LNP@BiTE‐mRNA had a higher tumor inhibition rate (70%) than BiTE (50%) (Figure 7F). Meanwhile, mouse weights from each treatment group did not change significantly during the course of treatment (Figure 7G). Similarly, the tumor individual or total bioluminescence intensity also showed the same trend as the tumor bioluminescence imaging graph (Figure 7H,I). We also monitored tumor growth by bioluminescence at 8 and 26 days. (Figure 7J). On day 26, the smallest fluorescence intensity was observed in mice treated with LNP@BiTE‐mRNA, followed by tumors treated with BiTE. This is consistent with the human hematological tumor xenograft model experimental results.

### 2.9 LNP@BiTE‐mRNA Increased T cell Infiltration in a Melanoma Subcutaneous Tumor Model

**Figure 8:**
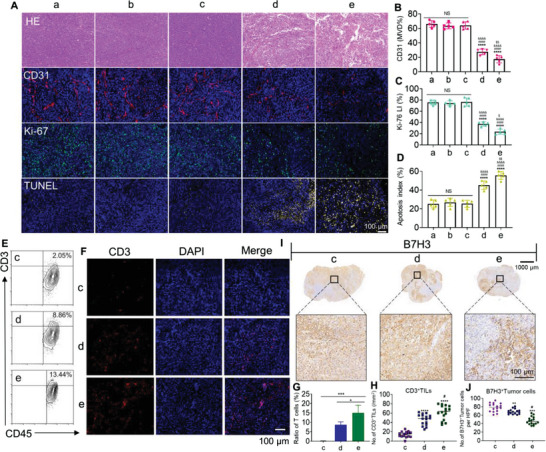
In vivo lymphocyte infiltrate and histology of tumors assessment in human melanoma subcutaneous tumor models. A) H&E staining, CD31, Ki‐67, and TUNEL immunofluorescent staining of tumor slices. Scale bars, 100 µm. B–D) Microvessel density (MVD), mean Ki‐67 LI, and mean apoptotic index in each group. ****p < 0.0001 versus the group of (a). ^####^ *p* < 0.0001 versus the group of (b). ^&&&&^ *p* < 0.0001 versus the group of (c). ^$^ *p* < 0.05 and ^$$^ *p* < 0.001 versus the group of (d). *n*  = 5. E,F) Tumor‐infiltrating T cells (CD3+) were quantified and determined by using FACS and fluorescence IHC. Scale bars, 100 µm. G,H) CD3^+^ TILs from intact tumors from the (c), (d), and (e) groups were quantified by FACS and IHC. ****p < 0.0001 versus the group of (c). *p < 0.05 and ^#^ *p* < 0.05 versus the group of (d). I,J) B7H3‐expressing tumor cells were quantified by IHC in three consecutive tumor sections. Top, representative IHC images, Scale bars, 1000 µm. Arrowheads indicate positive staining. Scale bars, 100 µm. **p < 0.01 and ***p < 0.001 versus the group of (c). ^#^ *p* < 0.05 versus the group of (d). (a) Normal saline + T cell, (b) 22 mg kg^−1^ IC8‐LNP + T cell, (c) 1.5 mg kg^−1^ BiTE mRNA + T cell, (d) 6 mg kg^−1^ BiTE + T cell, (e) 1.5 mg kg^−1^ LNP@BiTE‐mRNA + T cell.

H&E staining, CD31, Ki‐67, and terminal deoxynucleotidyl transferase‐mediated deoxyuridine triphosphate nick end labeling (TUNEL) analysis were used to further evaluate the antitumor efficacies according to our previous study.^[^ ^27^ ^]^ H&E staining showed irregular structures in both the BiTE and LNP@BiTE‐mRNA treatment groups (**Figure** **8**A). The microvessel density (MVD) in the LNP@BiTE‐mRNA group was significantly decreased, with a lower MVD of ≈17.2 ± 2.03% than that in the BiTE group (29.2 ± 2.43%), BiTE‐mRNA group (74.0 ± 2.19%), IC8‐LNP group (72.0 ± 2.32%), and normal saline group (73.0 ± 2.08%) (Figure 8B). IHC staining of Ki‐67 was also performed (Figure 8C). As shown, the Ki‐67 LI of the LNP@BiTE‐mRNA group (26.5 ± 2.6%) was lower than that of the BiTE group (38.2 ± 2.2%). There were no substantive differences across the remaining three groups. Furthermore, the TUNEL assay (Figure 8D) suggested that the apoptotic index in the LNP@BiTE‐mRNA group was 60.4 ± 3.2%, which was higher than that in the BiTE group (43.3 ± 3.4%). Taken together, these results suggested that LNP@BiTE‐mRNA induced significant suppression of tumor growth and angiogenesis, with enhancement of apoptosis and necrosis of tumors. To assess whether activated T lymphocytes were recruited to the tumor site after treatment, we next analyzed the frequency of tumor‐infiltrating lymphocytes (TILs). We first detected the expression of CD3 in tumors by using FACS and fluorescence IHC after BiTE‐mRNA + T cells, BiTE + T cells, or LNP@BiTE‐mRNA + T cells treatment (Figure 8E,F). By FACS, we found that the LNP@ BiTE‐mRNA treatment group exhibited a relatively high ratio (13.44%) of CD3^+^ TILs compared with the BiTE treatment group (8.86%) and the most significant the mean fluorescence intensity (Figure 8E,G). the gating strategies used for flow cytometry analysis were performed as described in Figure S8, Supporting Information. Similarly, the LNP@BiTE‐mRNA group showed more clustering of the red small dots, indicating that T cells swarm around a tumor cell (Figure 8F) and the highest percentage of T cells (Figure 8H). In addition, IHC staining also showed B7H3 protein expression differences for the mentioned treatment groups. The most pronounced downregulation of B7H3 was observed after LNP@BiTE‐mRNA treatment (Figure 8I,J). This again validated the significant enhancement of the therapeutic efficacy of the LNP@BiTE‐mRNA.

## 3 Discussion

In recent years, increasing interest has been seen in using LNPs to deliver mRNA therapeutics. However, the key to mRNA therapeutics is itself and the delivery system. Since mRNA is easily degraded^[^ ^30^ ^]^ and stimulates the innate immune system, mRNA is very susceptible to many aspects, such as gene sequence modification, delivery systems, and production processes. The in vivo translation efficiency of mRNA molecules can be further increased by RNA engineering. To achieve effective translation, mRNA requires some structural elements, including the 5′ cap, 3′ poly(A) tail, protein‐coding sequence, nucleoside modification and 5′ and 3′ UTRs. The sequences of these elements regulate translation initiation, translation termination and posttranscriptional modification of mRNA molecules.^[^ ^9^, ^10^ ^]^ Thus, sequence engineering of these elements can improve translation in vivo. In this study, UTP was replaced by N1‐Me‐Pseudo UTP to obtain a large number of modified mRNAs. It can greatly inhibit host immune responses.^[^ ^31^, ^32^, ^33^ ^]^ However, naked mRNA molecules are easily degradable. Thus, rational design and optimization of the mRNA delivery system are crucial to overcome this obstacle.

Generally, mRNA‐LNPs formulations encapsulate four components. They are typically composed of ionizable cationic phospholipids, an assistant lipid such as distearoylphosphatidylcholine (DSPC), cholesterol, and polyethyleneglycol‐modified phospholipids respectively.^[^ ^34^ ^]^ The most prominent excipient is the ionizable cationic phospholipid that tends to be more positive and electrostatic interactions with negatively charged mRNA takes place, which is a decisive factor in mRNA delivery and transfection efficiency. A number of nanoparticle carriers^[^ ^1^, ^2^ ^]^ have been proposed to increase the delivery efficiency of mRNA, protecting the mRNA against degradation by ubiquitous RNases and assisting in transfection of the intended target cells.^[^ ^35^, ^36^ ^]^ In this work, the pH‐sensitive ionizable lipid IC8 prepared by a simple one‐step reaction has the advantages of rapid synthesis and controlled quality. It is beneficial to mRNA delivery in vivo since neutral lipids have less interaction with blood cells and less serum protein absorption. In this study, the mRNA loaded by the nanocrystals prepared by microfluidic technology initiated a robust protein expression in vivo and in vitro, which may be closely related to the hydroxyl group of IC8 that can flexibly adjust the lipophilicity of the preparation and reduce the adsorption of serum protein.^[^ ^37^ ^]^ In addition, when the preparation is trapped in endosomes, in which the pH is lower than that in the extracellular environment, ionizable lipids promote membrane destabilization and facilitate endosomal escape,^[^ ^38^ ^]^ which is probably due to the four ionizable N atom centers of IC8 that can flexibly adjust the proton sponge effect. Apart from that, according to the package insert, both Pfizer/BioNTech and Moderna COVID‐19 vaccines must be stored at ultralow temperature and should be discarded after less than a day at room temperature. Therefore, the storage stability of mRNA is a great challenge in the development of mRNA drugs. In this study, we investigated the storage stability and transfection stability of LNP@GFP‐mRNA and found that it could be stably stored at 4 °C for one month, and the transfection efficiency of LNP@GFP‐mRNA did not significantly decrease within one month. To further optimize the stability of RNA, circular RNA is considered a promising alternative.^[^ ^39^, ^40^ ^]^ CircRNA lacks the free ends necessary for nuclease‐mediated degradation and possesses a covalent closed ring structure, which is proven to mediate potent and durable protein expression in vivo and has a longer half‐life than its linear mRNA counterpart.^[^ ^39^ ^]^ This should be taken into account in our subsequent studies.

Another significant factor was the serum half‐life of the antibody encoded by mRNA, which is determined by the half‐life of the mRNA encoding the antibody on the one hand and the antibody itself on the other hand. Many BiTE have been engineered by linking antibody fragments, such as single‐chain variable fragments (scFv), antigen‐binding fragments, and heavy (VH) and light chain (VL) variable domains, as well as their appendages to IgG‐format mAbs.^[^ ^41^, ^42^, ^43^ ^]^ However, these novel formats, deviating from the conventional IgG structure, often suffer from poor physicochemical properties. Poor solubility, short half‐life, and require repeated doses result in limitations on clinical application to achieve its therapeutic activity.^[^ ^43^ ^]^ In this article, our BiTE is structurally similar to Blinatumomab, which been previously reported.^[^ ^44^ ^]^ Blinatumomab, a CD19/CD3 BiTE designed in the BiTE (bispecific T‐cell engager) format, is approved by the US Food and Drug Administration for the treatment of relapsed or refractory B‐cell precursor AML. Because it can be quickly cleared by the kidney during circulation, blinatumomab has a short half‐life of 2 h and requires continuous intravenous dosing. Therefore, we designed a novel strategy by using LNP@mRNA platform technology to optimize the half‐life of short‐lived proteins for this type of antibody structure.^[^ ^45^, ^46^ ^]^ Notably, in our study, such an mRNA‐delivery route offers the ability to lower the administered dose (1.5 mg kg^−1^) while achieving a satisfactory therapeutic effect. Compared to previously published studies.^[^ ^4^, ^47^ ^]^ We observed relatively higher protein expression and longer antibody persistence in mouse serum after delivery of LNP@BiTE‐mRNA. Besides, the most common fusion protein technology are Fc fusion proteins, serum albumin fusion proteins, and cytoplasmic transduction peptide ‐fused protein, which can prolong the half‐life through FcRn‐mediated circulatory pathway and increase the molecular size.^[^ ^48^ ^]^ if the protein fusion technology is applied to the mRNA‐LNPs strategies, its physicochemical properties will be further improved, which is the direction we want to study next.

It is worth discussing the different in vivo therapeutic efficacies in the two tumor models. Excellent experimental results showed that LNP@BiTE‐mRNA can also achieve better therapeutic outcomes in AML‐than in solid tumors. This is in good agreement with that reported in the literatures.^[^ ^49^ ^]^ As reported by Hannah et al., they developed a novel CD19/CD3 BiTE in the single‐chain Fv–Fc format (CD19/CD3‐scFv‐Fc) with a half‐life of ≈5 days. Overall, prolonging the half‐life of BiTE enhances its therapeutic efficacy. Their study concept and design are in line with our purpose of BiTE modification. Furthermore, there have been clinical case studies reports of acute liver failure caused by hematologic malignancies.^[^ ^50^ ^]^ Pardi et al. already showed that the intravenous injection of mRNA‐LNPs leads to robust protein expression in the liver.^[^ ^51^ ^]^ Our data showed that there was a lower number of metastases in the LNP@BiTE‐mRNA treatment group, suggesting that LNP@BiTE‐mRNA could significantly inhibit MV411 cancer metastasis to the liver. Meanwhile, B7H3 expression level was obviously downregulated following LNP@BiTE‐mRNA treatment. These findings suggest that our strategy can potentially enhance therapeutic efficacy and reduce off‐target effects. Nevertheless, this improvement did not achieve satisfactory results in the treatment of solid tumors. The cause of incomplete solid tumor regression may be partly due to the acidic microenvironment of most solid tumors.^[^ ^52^ ^]^ However, some recent findings deserve attention. As seen with the published A Phase I/II clinical data of CLDN6 CAR‐T cells and CARVac from BioNtech at the aacr Meeting 2022,^[^ ^53^ ^]^ CLDN6 CAR‐T cells ± CARVac show a favorable safety profile at doses tested and encouraging signs of efficacy on solid tumors. The above results indicate that mRNA therapeutics has tremendous potential in a variety of tumors and in the field of tumor immunotherapy.

## 4 Conclusion

B7H3 belongs to the B7‐CD28 pathway and is considered to be a checkpoint molecule that has been found in a variety of cancers, such as melanoma and AML, and is associated with poorer clinical outcomes or more advanced disease in these patients. Here, mRNA encoding BiTE (an antibody that bispecifically binds and neutralizes CD3 and B7H3) was encapsulated in novel ionizable lipid nanoparticles targeting the liver. We confirmed the clear advantage of the LNP@mRNA, which could efficiently express BiTE in the desired organ and quickly translate into BiTE in mice. More importantly, a single intravenous injection of LNP@BiTE‐mRNA can consistently express antibodies within one week and its antibody half‐life is much greater than the half‐life of recombination BiTE, which can elicit robust and durable antitumor efficacy against hematologic and solid tumors. In addition, we also discovered that the LNP@BiTE‐mRNA we constructed with efficient had mRNA delivery performance and could be stably stored at 4 °C for one month, which might be attributed to the novel ionizable lipid IC8 synthesized through simple chemical reactions. Overall, our study was the first to use the mRNA‐LNP platform to generate therapeutic B7H3×CD3 BiTE in the host and achieved promising therapeutic effects. In addition, we also reported a novel ionizable lipid with great potential in mRNA delivery. Thus, we believe that our findings will serve as the basis for the use of the nucleoside‐modified mRNA‐LNPs platform for the delivery of B7H3×CD3 BiTE as well as other therapeutic antibodies and protein therapies adopted for tumor immunotherapeutic strategies.

## 5 Experimental Section

### Animals

8‐10‐week‐old immunodeficient male and female NSG (NOD‐PrkdcscidIL2rgem1/Smoc) mice were purchased from Beijing Huafukang Biotechnology Co. Ltd (Beijing Huafukang Biotechnology Co. Ltd, Beijing, China) and had a body weight of 18–22 g. Mice were housed up to five per cage and the cage bedding, feed and water were changed every three days for the duration of the experiment. All mice were maintained in a specific pathogen free environment at Sichuan University and were carried out in accordance with protocols approved by the Biomedical Research Ethics Committee at West China Hospital (Ethical approval document: 2018–061). All efforts were made to minimize suffering.

### Cell Culture

The human tumor cell lines A375, SKOV3, HeLa, HepG2, A549, Du145, U87, SW480, T24, MV411, THP‐1, U937, U266, Daudi, Raji, Jurkat, Molm13, mouse normal hepatocytes (AML‐12 cells), and human normal liver cells (LO2) were purchased from American Type Culture Collection (ATCC) and maintained in Dulbecco's modified Eagle's medium (DMEM; GIBCO), supplemented with 10% fetal bovine serum (FBS; GIBCO), 2 mmol L^−1^ l‐glutamine (Invitrogen), and 1× penicillin‐streptomycin (100 U mL^−1^ penicillin and 100 µg·mL^−1^ streptomycin). at 37 °C. AML‐12 cells were cultured in DMEM: F12 medium (PM150312) supplemented with 10% FBS (164210‐500), 10 µg mL^−1^ insulin, 5.5 µg mL^−1^ transferrin, 5 ng mL^−1^ selenium, 40 ng mL^−1^ dexamethasone and 1% P/S (PB180120). Cells in a logarithmic growth phase were used for all in vitro and in vivo experiments in 5% CO2.

### Preparation of Human Peripheral Blood Mononuclear Cells

Human peripheral blood mononuclear cells (hPBMCs) were isolated from the peripheral blood of healthy donors (informed written consent from all participants was obtained prior to the research) using density gradient separation (800 × *g* for 15 min at room temperature). The experiment was reviewed by the Biomedical Research Ethics Committee at West China Hospital (Ethical approval document: 2018–061). Lymphocyte separation solution (TBD, Tianjin, China) was used to isolate lymphocytes from whole blood, which were suspended in X‐vivo medium (Lonza). A total of 1× 10^6^ hPBMCs per mL of a 6‐well plate containing 2 mL of X‐vivo medium supplemented with 10% FBS (inactivated by heating to 56 °C for 30 min), l‐glutamine (2 mm, Gibco), 100 U mL^−1^ penicillin and 100 µg mL^−1^ streptomycin. hPBMCs were stimulated with an anti‐CD3 monoclonal antibody (OKT3, 200 ng mL^−1^, BioLegend), or anti‐CD28 monoclonal antibody (mAb) (CD28.2, 100 ng mL^−1^, BioLegend) for two consecutive stimuli. Meanwhile, the hPBMCs were continuously cultured with recombinant human IL‐2 (100 units mL^−1^, Life Science) to maintain optimal proliferation throughout the experiment.

### The Synthesis of Delivery Vehicles (IC8)

Appropriate amounts of 3‐[4‐(3‐aminopropyl)piperazin‐1‐yl]propan‐1‐amine and R)‐(+)‐1,2‐epoxytetradecane were dissolved in isopropyl alcohol (molar ratio of 3‐[4‐(3‐aminopropyl)piperazin‐1‐yl]propan‐1‐amine to R)‐(+)‐1,2‐epoxytetradecane was 1:5), stirred in a magnetic stirrer, and refluxed at 90 °C for 24 h. After the reaction, the products were collected by silica gel column chromatography (MeOH/DCM = 10:1).

### Formulation of mRNA‐Loaded LNPs

To synthesize LNPs, a microfluidic device was used to mix the aqueous phase containing mRNA with the ethanol phase containing lipids and cholesterol. Briefly, the aqueous phase was prepared with 13.5 mm citric acid buffer (pH = 3) and mRNA. The ethanol phase was prepared by mixing IC8, DSPC, cholesterol, and DMG‐PEG‐2000 with molar ratios of 35%, 16%, 46.5%, and 2.5%, respectively. The aqueous phase and ethanol phase were mixed in a 3:1 ratio in a microfluidic device. After mixing, the LNPs were dialyzed with 10 mm citric acid buffer at pH = 6 for 2 h and then sterilized with a 0.22 µm filter.

### mRNA‐LNPs Characterization

The average particle size, PDI, and zeta potential of these mRNA‐LNPs were measured by a Zetasizer Nano ZS90 (Malvern Instruments, Malvern, United Kingdom). Data were obtained as an average of three repetitions on different samples. The morphology of mRNA‐LNPs was identified using a transmission electron microscope (HT7800, Hitachi, Japan) after dropping samples onto a carbon‐formvar copper grid and negative staining with 2% phosphotungstic acid solution.

### Gel Retardation Assay

A gel electrophoresis retardation assay was performed to evaluate the mRNA‐binding ability of the complexation of mRNA and LNP at different time points. Free mRNA (0.5 µg) and LNP@GFP‐mRNA (containing 0.5 µg mRNA) were diluted with RNase‐free water. The running gel parameters were set to 120 for 20 min, and this electrophoresis process was stopped until the indicator reached 2/3 of the electrophoresis gel. The results were analyzed using a ChemiDocTM 219 XRS system (Gel Doc 2000, Bio–Rad Laboratories, Hercules, USA).

### LNP@GFP‐mRNA Stability

To study LNP@GFP‐mRNA storage stability at room temperature, the average particle size, and zeta potential were monitored for five weeks.

### Cell Transfection and Uptake Studies

Normal mouse liver AML‐12 cells, human normal liver cells (LO2) and HEK‐293T cells were inoculated in 24‐well culture plates (1 × 10^4^ cells per well) supplied with 0.5 mL DMEM (containing 10% FBS) for 24 h before transfection. Lipo8k was included as a positive control and the different groups were set to a medium containing no serum and 10% serum. Both lipo8K‐mRNA and LNP@GFP‐mRNA containing 1 µg GFP‐mRNA were added to 24‐well plates for 24 h incubation at 37 °C. Finally, inverted fluorescence microscopy (Nikon, Japan) and FACS (ACEA Bioscience) were used to evaluate the transfection effect. For the transfection stability test, 293T cells were transfected with LNP@GFP‐mRNA, at all sampled time points within one month.

### The Lysosome Escape Assay

The localization of LNP@CY5‐mRNA in AML‐12 cells was observed by CLSM (Zeiss Company, Oberkochen, Germany). AML‐12 cells (1 × 10^6^) were seeded on eight‐chamber coverglass for 24 h to allow them to attach to the bottom of the dish and incubated with LNP@CY5‐mRNA (APExBIO, R1009. The final concentration of CY5‐mRNA was 0.5 µg per chamber). After incubation, 2, 4, and 6 h, the cells were washed twice with precooled PBS, and lysosome were labeled by incubation with Lyso‐Tracker green (Beyotime, C1047S. 75 µm final concentration) one and half an hour before the termination of uptake. Thereafter, the cells were fixed with 3% glutaraldehyde for 15 min and stained with DAPI for observation.

### Construction of B7H3×CD3 BiTE

The VL and VH sequences for the anti‐B7H3 single‐chain variable fragment (scFv) sequence  were derived from a highly specific mAb against B7H3 (clone mAb‐J42) that has been reported in the lab group's previous studies.^[^ ^27^, ^54^, ^55^, ^56^ ^]^ DNAs encoding CD3‐specific single‐chain variable fragments (scFvs) were synthesized by Genewiz (https://www.genewiz.com) according to previously published amino acid sequences.^[^ ^27^ ^]^ The two scFvs (B7H3‐specific scFv and CD3‐specific scFv) were linked by a 5‐amino‐acid (G4S) linker to form a recombinant single‐chain BiTE. For recombinant BiTE expression in mammalian cell lines, cDNA encoding CD3‐specific scFv and B7H3‐specific scFv (J42‐scFv) was subcloned into a eukaryotic expression vector with a His tag at the C‐terminus to facilitate protein purification.

### Expression of Recombinant BiTE

The recombinant plasmids were exogenously expressed in HEK 293T cells that were transiently transfected with the expression vectors described by using Lipofectamine 3000. At 12 h posttransfection, transiently transfected cells were cultured in FreeStyle serum‐free medium (Thermo Fisher Scientific) at 37 °C with 5% CO2 in a humidified incubator, and supernatants were harvested 7 days after expansion. The supernatant was collected and filtered through a 0.22 µm filter membrane. Then, the recombinant B7H3×CD3 BiTE was affinity‐purified using Ni‐NTA affinity columns (GE Healthcare) and was gel filtered by using a Superdex 200 Increase 10/300 GL column (GE Healthcare). Purified BiTE was routinely analyzed by SDS‐PAGE and stained with Coomassie brilliant blue for size estimation and quality control.

### In Vitro Transcription and Purification of mRNA

In vitro transcription template generated by PCR based on the sequence from the B7H3×CD3 plasmid. RNA was produced following the protocol of the T7 High Yield RNA Transcription Kit (N^1^‐Me‐Pseudo UTP, Vazyme, DD4202). After in vitro transcription, the DNA templates were removed by digestion with RNase‐free DNase I (0.1 units µL^−1^) which was incubated at 37 °C for 15 min. The PCR product for in vitro transcription was further purified by precipitation with 8 m lithium chloride (Beyotime, ST498‐100 mL). RNA was quantified by using a NanoDrop‐1000 and RNA integrity was checked by electrophoresis.

### Vaccinia Capping System and *E. coli* Poly(A) Polymerase

The purified mRNA was then capped using a Vaccinia Capping System and purified again using LiCl precipitation. Briefly, the 5′ end of the mRNA was capped by the Vaccinia Capping system (Vazyme, DD4109) and then methylated by 2″‐O‐methyltransferase (Vazyme,DD4110) to obtain Cap1 RNA. Finally, the use of *E.coli* poly (A) polymerase (Vazyme,DD4111) can add 20–200 bases to the 3″ end of the mRNA, and after purification again, the mRNA structure with good stability and high translation efficiency can be obtained. All of the above procedures were performed according to the manufacturer's instructions.

### Lentivirus Transfection

To establish the A375‐Luc and MV411‐Luc cell lines, lentiviruses (luciferase viruses) were used to infect A375 and MV411 cells. Briefly, luciferase plasmids were co‐transfected with packaging plasmids (PSPAX2 and PMD2‐G) into 293T cells to produce virus, A375 and MV411 cells were infected with the viruses and selected with 3 µg/ml puromycin for 2 days, followed by 14 days of maintenance in 1 µg mL^−1^ puromycin. Then, A375‐Luc and MV411‐Luc stable cell lines were finally obtained.

### Flow Cytometry

The expression of B7H3 on tumor cell lines and tumors from mice was determined by FACS. B7H expression was first examined in a variety of tumor cell lines including A375, SKOV3, HeLa, HepG2, A549, Du145, U87, SW480, T24, MV411, THP‐1, U937, U266, Daudi, Raji, Jurkat, Molm13, and human normal liver (LO2) cells. Second, Tumor‐bearing mice were anesthetized, and tumor tissues were removed after treatment termination. Tumor tissues were finely minced with scissors and digested (collagenase: 1 mg mL^−1^) for 30 min at 37 °C to prepare a single‐cell suspension. The tumor single‐cell suspension was analyzed for CD3 expression using a BD Fortessa cytometer and analysis was performed using FlowJo software. Specifically, cells were incubated with a total of 10 µL of three‐color antibodies, Fixable Viability Dye (Thermo Fisher 65 086 618), CD45 (Proteintech FITC‐65109), and CD3 (Proteintech, PE‐65133) according to the manufacturer's instructions. Similarly, to detect B7H3‐positive MV411 cells in peripheral blood using FACS, mouse peripheral blood was obtained after removal of eyeballs after 22 days of inoculation and stained with the human B7H3 antibody (BioLegend, 331 605). Apart from this, to explore the impact of BiTE on T cell phenotype analyses, T cells were stained using the antibodies for anti‐human CD4 (BioLegend, 357 419) and anti‐human CD8 (BioLegend, 344 729) and analyzed by a Fortessa flow cytometer (BD).

### Cytotoxicity Assays In Vitro

2D tumor models with T cells were used to observe cell morphology and assess cytotoxicity. In the 2D model, target cells (A375, SKOV3, MV411 and THP‐1 cells) were cocultured with effector cells (T cells) at an E:T ratio of 5:1 together with 5 µg mL^−1^ BiTE. Target cells and effector cells were labeled with CFSE and Cyto Tell Red, respectively, prior to the cocultures. The observation was performed at 24 h after coculture under the different conditions. Meanwhile, based on the same methodology described, CFSE and Cyto Tell Red were used to represent the tumor and T cells, respectively, and FACS detection of the percentages of T cells and residual tumor cells (A375, MV411).

### Analysis of Cytokine Secretion

Target cells (A375, SKOV3, MV411, and THP‐1 cells) were cocultured with effector cells (E:T ratio, 5:1) in a 96‐well plate at 37 °C with the addition of 5 µg mL^−1^ BiTE. After 24 h, the supernatant was collected to analyze the tumor necrosis factor *α* (TNF‐*α*), interleukin (IL)‐2, and interferon *γ* (IFN‐*γ*) secretion from the effector cells using ‐enzyme linked immunosorbent assay (ELISA) kit (Thermo Fisher Scientific) according to the manufacturer's protocols.

### Real‐Time Cytotoxicity Assays

The cytotoxic ability of T cells with an addition of BiTE to lyse target cells was determined using the xCELLigence real‐time cells analyzer (ACEA Bioscience, Inc. xCELLigence RTCA SP). To start the real‐time cell analysis, background readings from 100 µL of media added to each well of the E‐plate 96 were obtained. For the cells to attach to the E‐plates, the A375 tumor cells (104 cells per well) were cultured in E‐plate 96 (ACEA Bioscience) for ≈15 h, and BiTE (5 µg mL^−1^) and T cell suspensions (E: T = 5:1) were added into the specific well. Three replicates were available for each well. Cell index measurements were performed at 15 min intervals for 72 h. The The real‐time cytotoxicity assays xCelligence system, based on microelectronic impedance technology, was used to assess cytotoxic ability. The size of impedance depends on the number, size, and shape of the adherent cells and the quality of the cell‐substrate attachment. The data were acquired and analyzed using the manufacturers´ protocols (ACEA Bioscience, Inc. RTCA Software 2.1).

### In Vivo Biodistribution of LNP@Luc‐mRNA and Protein Expression of B7H3×CD3 BiTE

A time course biodistribution of the luciferase activity was conducted using an in vivo imaging system (Caliper Life Sciences) according to the manufacturer's protocols. In vivo expression of the luciferase mRNA transfection was detected in 8‐10‐week‐old immunodeficient male NSG, with three mice in each group. After the injection of LNP@Luc‐mRNA, mice were photographed at different time intervals for 72 h and luciferase activity was determined by a luciferase imaging system. Mice were injected intraperitoneally with a 150 mg kg^−1^ dose of d‐luciferin potassium salt (MeiLunBio) before imaging. Mice were euthanized at 6 h after the injection, and luciferase activity was assessed in the heart, liver, spleen, lung and kidney using the Luciferase Assay System. The average radiance of the region of interest (ROI) was measured by Living Image 4.3.1 Software (https://www.perkinelmer.com).

For B7H3×CD3 BiTE protein expression in vivo, the serum was collected at different time points after a single injection of BiTE and LNP@BiTE‐mRNA. The BiTE concentration was determined via ELISA. For detection of B7H3×CD3 BiTE, CD3*ε* and CD3*γ* Protein (Kactus Biosystems, CD3‐HM257) were coated overnight at 4 °C with a human anti‐idiotype antibody to capture the CD3 scFv region. The wells were washed three times in PBST, blocked at room temperature with 1% BSA in PBS for 2 h, and again washed three times in PBST. The standard curve was made with a serial dilution of the corresponding purified recombinant B7H3×CD3 BiTE in vitro. NSG mouse plasma was used as the primary antibody. The wells were incubated with primary antibody for 1 h and the anti‐6×His‐HRP secondary antibody (1:1000) for 1 h at room temperature. Following a final wash, the plates were treated with TMB substrate solution (SeraCare) at room temperature in the dark for 15 min. The reaction was stopped by the addition of 2.5 m H_2_SO_4_ and read at 450 nm using a microplate reader (Bio Tek microplate reader). The approach of luciferase activity quantification varied slightly but was approximately as follows. Briefly, mice were sacrificed at different time point intervals for 72 h as indicated after the injection of LNP@Luc mRNA, and the livers were removed. Next, mouse liver tissue samples were freeze‐dried, ground and prepared using a radio‐immunoprecipitation (RIPA) buffer containing protease inhibitor cocktail mix and 5 mm EDTA. The cell lysates were centrifuged, and the supernatant proteins were collected. A luciferase assay kit was used to measure the Luc protein concentration according to the manufacturer's protocols.

### Animal Studies and Bioluminescent Imaging

Two types of tumors were selected for this animal study. In the hematologic tumor model, 2 × 10^6^ MV411‐Luc leukemia cells were injected into the tail vein. In the melanoma subcutaneous tumor xenograft model, 2 × 10^6^ A375‐Luc cells were subcutaneously injected into female NSG mice. Cells were collected in logarithmic growth phase and inoculated into mice as soon as possible. After tumor inoculation, mice were intravenously injected with 1 × 10^7^ T cells and 100 U IL‐2 in 100 µL of PBS via the tail vein on days 3, 10, and 17. From the eighth day on, the BiTE‐mRNA (1.5 mg kg^−1^), BiTE (6 mg kg^−1^), and LNP@BiTE‐mRNA (1.5 mg kg^−1^) treatment groups in combination with 1 × 10^7^ T cells and 100 U IL‐2 were intravenously administered. Every three or four days, tumor sizes were measured by a slide caliper and mouse bodies and subcutaneous tumors were weighed (HZT, USA). The tumor volume was calculated by the formula: Volume = Length×Width×Height /2.

The tumor‐bearing mice were photographed after inoculation using an in vivo imaging system (Caliper Life Sciences). Bioluminescent imaging (BLI) was performed as described previously. All mice were euthanized immediately after the last in vivo BLI. Tumor tissues and main organs were harvested and photographed followed by immediate fixation using 10% formalin solution and paraffin embedding.

### Immunofluorescence and IHC Staining

A375, SKOV3 and HeLa cells were seeded onto eight‐chamber coverglass (Cellvis, C8‐1.5 h‐N) at a density of 1× 10^4^ cells mL^−1^ and incubated at 37 °C in a 5% CO2 incubator overnight. The cells were fixed with 4% paraformaldehyde and blocked with 5% BSA for 1 h. After blocking in 1% BSA, the cells were incubated with B7H3 (clone mAb‐J42) primary antibody for 1 h and then incubated with CoraLite594‐conjugated secondary antibody (1:500; Proteintech, 20 000 418) at room temperature in the dark for 45 min. Cell nuclei were stained with 4,6‐diamidino‐2‐phenylindole (DAPI) (Beyotime) and observed and photographed under an inverted fluorescence microscope (Olympus IX‐73). The suspended tumor cells (MV411, U937, and THP‐1 cells) were collected into a closed‐centrifuge tube. Then, similar procedures to those described above were conducted.

TMAs containing 40 cases of skin malignant melanoma, 30 adjacent normal skin tissues, and 10 normal skin tissues was purchased from Xi'an Alenabio and Shanghai Outdo Biotech of China and stained with an anti‐B7H3 antibody (CST 14058S). Next, tumor tissue was sectioned and subjected to IHC to detect CD31(Servicebio, GB11063), Ki67(Servicebio, GB13030‐2), and TUNEL (Servicebio, G1507) staining to analyze tumor cell proliferation, apoptosis, and angiogenesis. To evaluate T lymphocyte infiltration and the expression of surface proteins on tumor cells and tumor tissue specimens stained for CD3 (Servicebio, GB13014) and B7H3 (CST 14058S) were used. Meanwhile, the main organs of the mouse, including the heart, liver, spleen, lung, and kidney, were removed for HE staining. For the analysis of all markers, areas with pronounced inflammation or necrosis were avoided. In brief, to retrieve antigenicity, the tissue sections (3 mm thick) were first incubated at 65 °C for 1 h and then blocked with 10% serum for 1 h at room temperature. Subsequently, sections were incubated with primary antibody overnight at 4 °C and then incubated with secondary antibodies for 1 h at room temperature. The fluorescence IHC staining manipulation followed the same steps as the IHC assay. The only difference was that immunohistochemistry staining using fluorescently tagged secondary antibodies (Servicebio, GB213031) was performed. Pictures of stained sections were captured using a Digital Pathology System (Pannoramic MIDI, 3DHISTECH, Hungary).

### Statistical Analysis

The relationship between B7H3 gene expression and prognosis was performed using the melanoma dataset of the KM Plotter. B7H3 mRNA expression in the different stages of tumor progression of melanoma patients was examined by data mining in Cancer Genome Atlas (TCGA)‐melanoma using the UCSC xena browser (https://ucscxenabrowser.net). The meta‐analysis was performed by using Oncomine (www.oncomine.org). Boxplots with significance levels were plotted using the “ggpubr” package version 0.2.4 (http://127.0.0.1:21954/help/library/ggpubr/html/00 Index. html) in R language (version 3.6.1). The method of database analysis of human acute leukemia is the same as described above.

The results were expressed as standard deviations (SD). The unpaired t‐test with Welch's correction was used for the two‐group comparison. one‐way ANOVA with Holm–Sidak adjusted p values. Data were analyzed by two‐way ANOVA comparison between multiple groups. Non‐parametric or group comparisons with small sample sizes were assessed by unpaired two‐tailed Mann–Whitney U test. Overall survival was compared using the log‐rank (Mantel–Cox) test. Statistical analyses were performed using GraphPad Prism 8.0 and considered significant at *p < 0.05, **p < 0.01, ***p < 0.001, and ****p < 0.0001. NS: no significance.

## Conflict of Interest

The authors declare no conflict of interest.

## Authors Contribution

C.H., X.D., and J.W. contributed equally to this work. A.T, X.S, C.H, X.D, J.W, Q.T, Y.R, and K.C designed the study. Y.F analyzed the data from Oncomine and TCGA. C.H, X.D, J.W, Q.T, Y.R, Z.Z, Y.L, Y.F, Z.W, K.Z, Y.W, L.Z, and G.G performed the experiment and analyzed the data. C.H, X.D, X.S, and A.T wrote the manuscript. All authors read and approved the final manuscript.

## Supporting information

Supporting Information

## Data Availability

The data that support the findings of this study are available from the corresponding author upon reasonable request.
