# Supplementary material for: Lipid Nanoparticle Delivery System for mRNA Encoding B7H3‐redirected Bispecific Antibody Displays Potent Antitumor Effects on Malignant Tumors
Source: Adv Sci (Weinh). 2022 Nov 20;10(3):2205532. doi: 10.1002/advs.202205532 (PMC9875623; doi:10.1002/advs.202205532)
Supplement: Supplementary file 1 — Supporting Information [file ADVS-10-2205532-s001.pdf]

ADVANCED SCIENCE
Open Access

Supporting Information

for *Adv. Sci.*, DOI 10.1002/advs.202205532

Lipid Nanoparticle Delivery System for mRNA Encoding B7H3-redirected Bispecific Antibody Displays Potent Antitumor Effects on Malignant Tumors

Cheng Huang, Xing Duan, Jichao Wang, Qingqing Tian, Yangmei Ren, Kepan Chen, Zongliang Zhang, Yuanyou Li, Yunyu Feng, Kunhong Zhong, Yuelong Wang, Liangxue Zhou, Gang Guo, Xiangrong Song and Aiping Tong**

Supporting Information

Lipid Nanoparticle Delivery System for mRNA Encoding B7H3-redirected Bispecific Antibody Displays Potent Antitumor Effects on Malignant Tumors

Cheng Huang†, Xing Duan†, Jichao Wang†, Qingqing Tian, Yangmei Ren, Kepan Chen, Zongliang Zhang, Yuanyou Li, Yunyu Feng, Kunhong Zhong, Yuelong Wang, Liangxue Zhou, Gang Guo, Xiangrong Song, Aiping Tong **

†These authors contributed equally to the manuscript.

C. Huang, X. Duan, J. Wang, Q. Tian, Y. Ren, K. Chen, Z. Zhang, Y. Feng, K.Zhong, Prof. G. Guo

State Key Laboratory of Biotherapy and Cancer Center, Research Unit of Gene and Immunotherapy，Chinese Academy of Medical Sciences, Collaborative Innovation Center of Biotherapy, West China Hospital, Sichuan University, Chengdu, Sichuan Province, China.

Y. Li, M.D. Y. Wang, Prof. L. Zhou

Department of Neurosurgery, West China Hospital, West China Medical School, Sichuan University, Chengdu, Sichuan Province, China.

* Corresponding author. State Key Laboratory of Biotherapy and Cancer Center, Research Unit of Gene and Immunotherapy，Chinese Academy of Medical Sciences, Collaborative Innovation Center of Biotherapy, West China Hospital, Sichuan University, Chengdu, Sichuan Province, China.

Aiping Tong, Ph.D. E-mail address: aipingtong@scu.edu.cn

Address: No.17 Section 3, South Renmin Road, Wuhou District, Chengdu, Sichuan Province, China. Phone & Fax: +86-28-85502796.

* Corresponding author. Department of Critical Care Medicine, and Department of Pancreatic Surgery, Frontiers Science Center for Disease-related Molecular Network,

State Key Laboratory of Biotherapy and Cancer Center, West China Hospital, Sichuan University, Chengdu, Sichuan Province, China.
Xiangrong Song, Ph.D. E-mail address: songxr@scu.edu.cn (X. Song)
Address: No.17 Section 3, South Renmin Road, Wuhou District, Chengdu, Sichuan Province, China. Phone & Fax: +86-28-85502796.

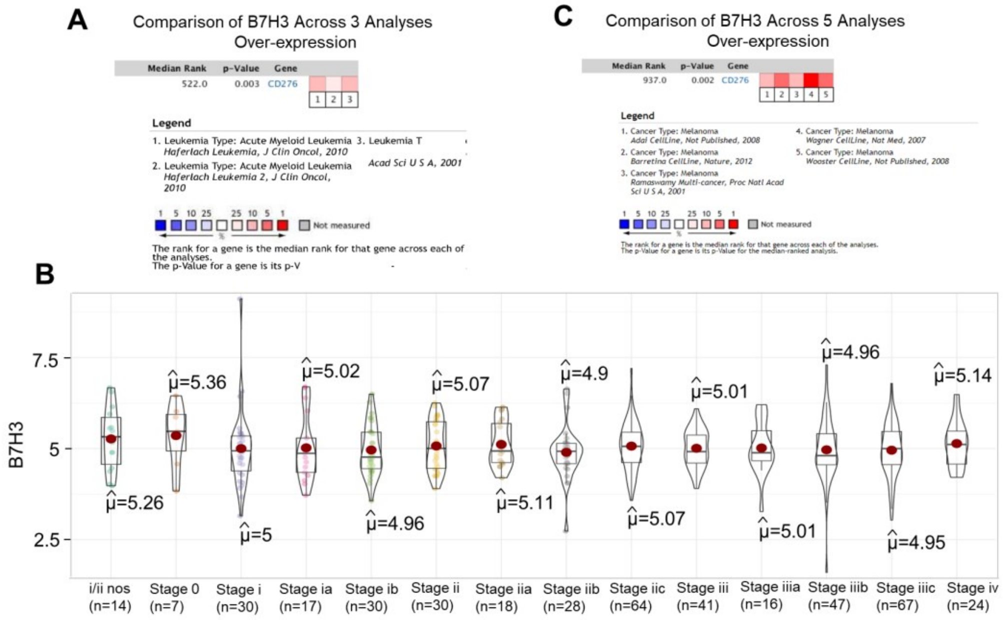

Figure S1. The B7H3 were subjected to expression analysis of AML and melanoma patient samples based on the database. A) Expression of B7H3 in AML in the studies derived from the Oncomine database. B) The expression level of B7H3 mRNA expression at different clinical stages (stage0-iv) in human melanoma patients (P>0.05). C) Expression of B7H3 in melanoma in the studies derived from the Oncomine database.

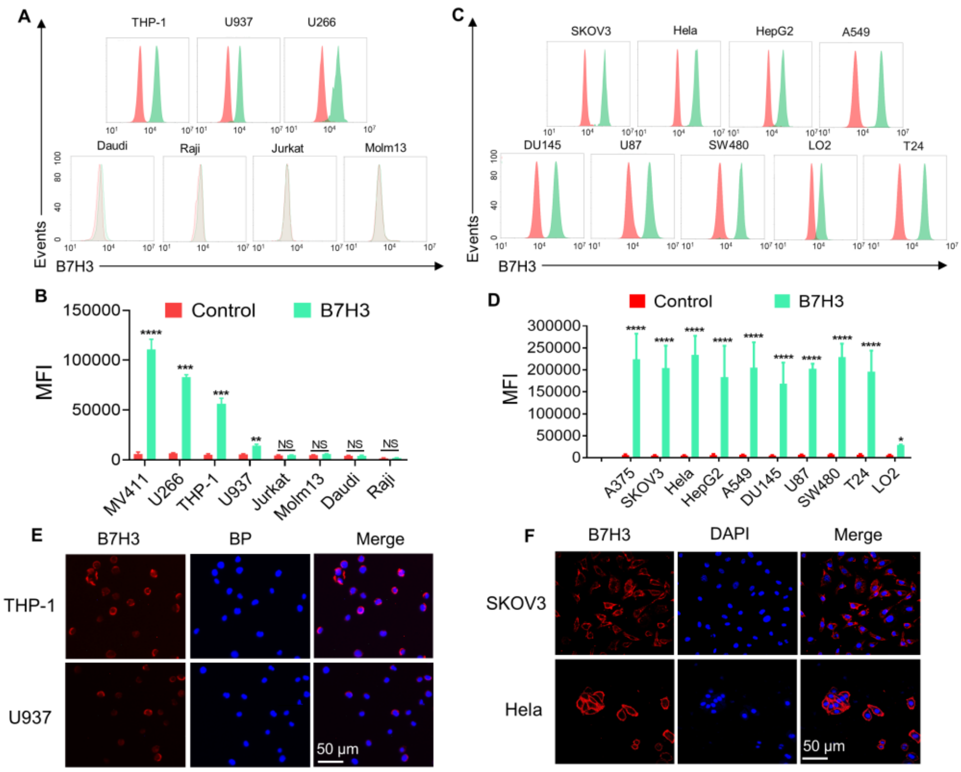

Figure S2. Expression of the costimulatory molecule B7H3 in various human tumor cell lines. A) B7H3 protein expression was examined in various human hematologic tumors cell lines by flow cytometric analyses. B) Histogram of mean fluorescence intensity in figure A. $^{**}P < 0.01$, $^{***}P < 0.001$, $^{****}P < 0.0001$. C) B7H3 protein expression was examined in various human solid tumor cell lines by flow cytometric analyses. D) Histogram of mean fluorescence intensity in figure C. $^{*}P < 0.05$, $^{****}P < 0.0001$ E, F) Immunofluorescence staining patterns showed high expression levels of B7H3 in different cancer cell lines (THP-1, U937, SKOV3, HeLa). Scale bar, 50 μm.

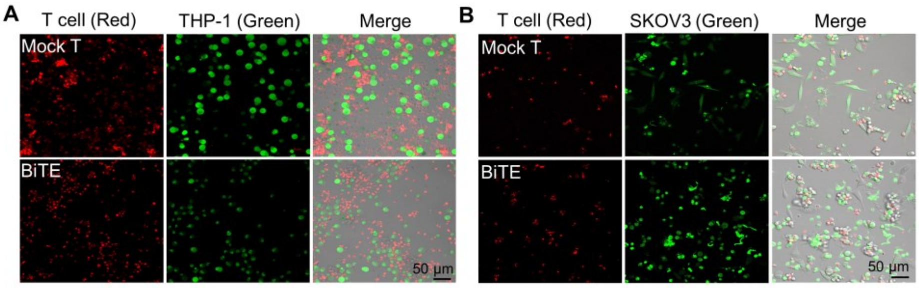

Figure S3. The morphology of tumor cell lysis was analyzed using confocal microscopy. Target cells THP-1 (A) and SKOV3 (B) and effector cells (T cells) were fluorescently labeled with CFSE and Cyto Tell Red, respectively (E:T=5:1). The observation was performed after 24 h' coculture under the different conditions.

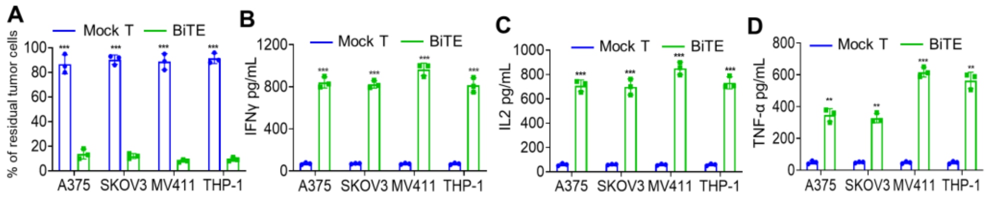

Figure S4. Anti-Tumor activity of BiTE in different types of tumor cell lines in vitro. A-D) After coculturing mock or BiTE-T cells with tumor cells at an E:T ratio of 5:1 for 24 h, the percentages of residual tumor cells were estimated from the FACS data (A), and the concentrations of IFN-g (B), IL-2 (C), and TNF-a (D) in supernatants were measured by ELISA kits. All error bars represent SD. **p < 0.01, ***p < 0.001.

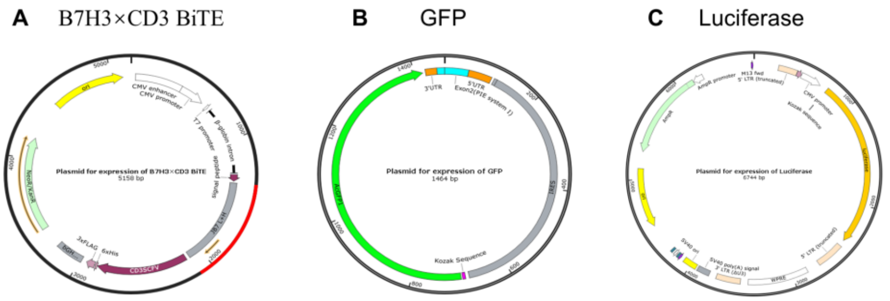

Figure S5. The expression plasmid map of B7H3×CD3 BiTE, GFP and luciferase. The sequences encoding B7H3×CD3 BiTE (A), GFP (B) or firefly luciferase (C) were subcloned into the pVAX vector. The corresponding DNA sequences in order are shown for the B7H3×CD3: (T7): TAATACGACTCACTATAGGG, (5'UTR): GAAATAAGAGAGAAAAGAAGAGTAAGAAGAAATATAAGAG CCACCTCTAG, (KOZAK): GCCACC, (5'F and signal peptide):ATGGAGTTTG GGCTGAGCTGGGTTTTCCTCGTTGCTCTTTTTAGAGGTGTCCAGTG, anti-B7

H3 (as described in our previous studies)[1], linker: GGTGGTGGTGGTAGCGGT GGTGGTAGCGGTGGTGGTGGTAGC, anti-CD3: GACATCAAGCTGCAGCAG TCAGGGGCTGAACTGGCCAGGCCTGGGGCTTCAGTGAAGATGTCCTGCAA GACCTCTGGCTACACCTTCACCAGATACACCATGCACTGGGTGAAGCAGAG GCCTGGACAAGGCCTTGAGTGGATCGGATACATTAACCCTTCTAGAGGCTA TACTAACTACAATCAAAAGTTCAAGGACAAGGCCACATTGACTACCGACAA GTCCTCCAGCACAGCCTACATGCAGCTCAGCAGCCTGACATCTGAGGACTC TGCGGTCTATTACTGTGCCAGATATTACGACGACCACTATTGCCTGGACTAC TGGGGCCAAGGCACCACGCTGACCGTCAGCAGCGTGGAGGGCGGTTCAG GCGGAAGCGGCGGGAGCGGTGGCAGCGGAGGCGTGGACGACATCCAGCT GACCCAGAGCCCAGCCATCATGAGCGCCAGCCCCGGCGAGAAGGTGACCA TGACCTGTAGGGCCAGCTCAAGTGTAAGTTACATGAACTGGTACCAGCAGA AGAGCGGTACCAGCCCAAAGAGATGGATCTACGACACATCCAAGGTGGCT TCTGGTGTGCCATACAGATTCAGCGGTAGCGGTAGCGGTACCAGCTACAGC CTCACCATCAGCAGCATGGAGGCTGAGGACGCCGCCACCTACTACTGCCA GCAGTGGAGTAGTAACCCACTCACGTTCGGCGCTGGGACCAAGCTG, (3’U TR): ACTTCCTACTCAGGCTTTATTCAAAGACCAAGAGGTACAGGTGCAAG GGAGAGAAGAAGGGCATGGCCAGAAGGCAAGCCCCGCAGAAG, (3’R)TTA CTTGTCATCGTCATCCTTGTAATC.

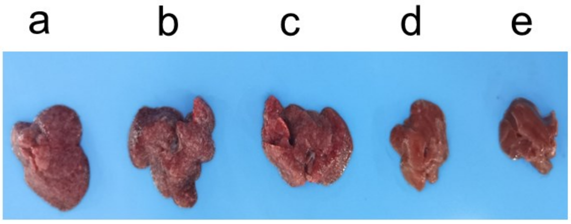

Figure S6. The liver photographs from MV411 tumor-bearing mouse on day 30 after the indicated treatments. a: Normal saline + T cell, b: 22 mg/kg IC8-LNPs + T cell, c: 1.5 mg/kg BiTE mRNA + T cell, d: 6 m/kg BiTE + T cell, e: 1.5 mg/kg BiTE mRNA-LNPs + T cell.

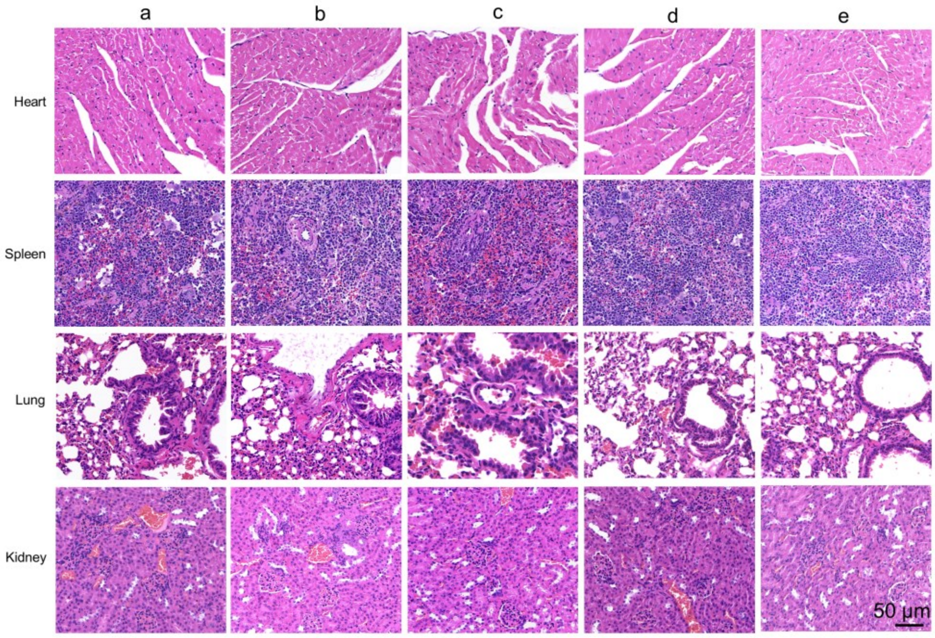

Figure S7. In vivo safety assessment of the the treatment groups was evaluated by H&E staining in human hematological tumor xenograft models. The pictures represent staining results of heart, spleen, lung and kidney. Scare bar, 50 μm. after the indicated treatments. a: Normal saline + T cell, b: 22 mg/kg IC8-LNPs + T cell, c: 1.5 mg/kg BiTE mRNA + T cell, d: 6 m/kg BiTE + T cell, e: 1.5 mg/kg BiTE mRNA-LNPs + T cell.

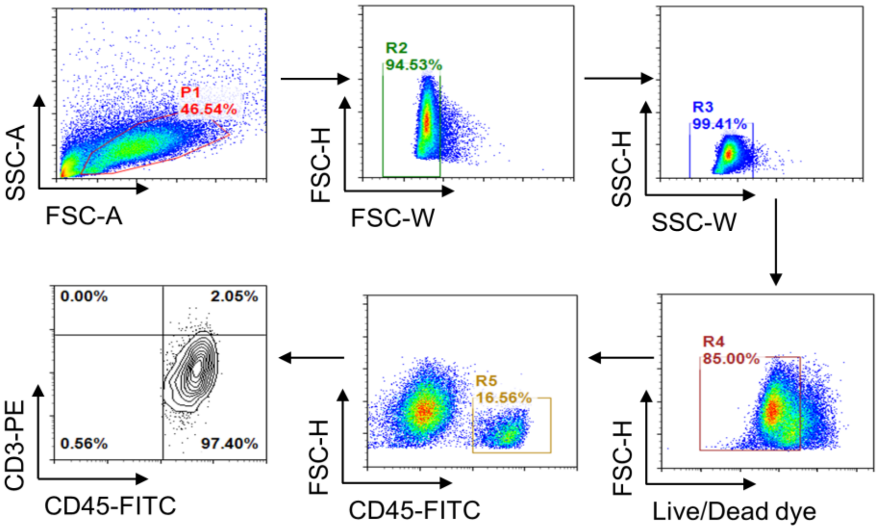

Figure S8. Gating strategies used for flow cytometry analysis of Tumor-infiltrating T-lymphocytes ($CD45^+$ $CD3^+$) in the melanoma tissues.

Table S1. Analysis of B7H3×CD3 BiTE pharmacokinetic parameter in mouse Serum.

Parameter	B7H3×CD3 BiTE mRNA-LNP (1.5 mg/kg)	B7H3×CD3 BiTE (6mg/kg)
T_{max}(hr)	6	1
Cmax(μg/mL)	6.455	6.251
AUC(hr*μg/mL)	146.6	37.4
$T_{1/2}$(hr)	74	2.3

Parameters were calculated using Phoenix pharmacokinetic software (Certara, USA), $T_{1/2}$, half-life; AUC_{0-t}, the area under the curve. C_{max}: maximum plasma concentration; T_{max}: time to reach Cmax.

[1] a) C. Huang, H. Li, Y. Feng, X. Li, Z. Zhang, C. Jiang, J. Wang, C. Yang, Y. Fu, M. Mu, S. Zhao, Z. Wang, Y. Kuang, H. Hou, Y. Wang, W. Guo, J. Xu, H. Yang, L. Zhou, A. Tong, G. Guo, *Theranostics* **2020**, 10, 10498; b) H. Li, C. Huang, Z. Zhang, Y. Feng, Z. Wang, X. Tang, K. Zhong, Y. Hu, G. Guo, L. Zhou, W. Guo, J. Xu, H. Yang, A. Tong, *Frontiers in oncology* **2020**,

10, 1527; c) Z. Zhang, C. Jiang, Z. Liu, M. Yang, X. Tang, Y. Wang, M. Zheng, J. Huang, K. Zhong, S. Zhao, M. Tang, T. Zhou, H. Yang, G. Guo, L. Zhou, J. Xu, A. Tong, *Mol Ther Oncolytics* **2020**, 17, 180; d) X. Tang, Y. Wang, J. Huang, Z. Zhang, F. Liu, J. Xu, G. Guo, W. Wang, A. Tong, L. Zhou, *Signal transduction and targeted therapy* **2021**, 6, 125.
